# Supplementary material for: Galangin: A Promising Flavonoid for the Treatment of Rheumatoid Arthritis—Mechanisms, Evidence, and Therapeutic Potential
Source: Pharmaceuticals (Basel). 2024 Jul 19;17(7):963. doi: 10.3390/ph17070963 (PMC11279697; doi:10.3390/ph17070963)
Supplement: Supplementary file 1 [file pharmaceuticals-17-00963-s001.zip › Table S1 Summary of the Chemical and Physical Properties of Galangin.pdf]

Table S1. Summary of the Chemical and Physical Properties of Galangin. National Center for Biotechnology Information (2024). PubChem Compound Summary for CID 6036, D-Galactose. Retrieved June 24, 2024 from https://pubchem.ncbi.nlm.nih.gov/compound/D-Galactose.

Property	Result/value
Common name	Galangin
Category	Flavonol
IUPAC name	3,5,7-trihydroxy-2-phenylchromen-4-one
Other names	Norizalpinin; teptochrysin; 3,5,7-Trihydroxyflavone; 3,5,7-triOH-Flavone; 3,5,7-Trihydroxy-2 phenyl-4-benzopyrone
Canonical SMILES	C1=CC=C(C=C1)C2=C(C(=O)C3=C(C=C(C=C3O2)O)O)O
Molecular formula	$C_{15}H_{10}O_5$
Molecular weight	270.24 g/mol
Hydrogen Bond Donor	3
Hydrogen Bond Acceptor	5
Rotatable Bond	1
XLogP3	2.3
Topological polar surface area	87Å^2
Percent composition	C = 66.67%, H = 3.73%, O = 29.6%
Exact Mass	270.05282342 g/mol
Monoisotopic Mass	270.05282342 g/mol
Heavy atom count	20
Formal Charge	0
Complexity	424
Isotope Atom Count	0
Atom Stereocenter count	0
Bond Stereocenter count	0
Covalently bonded unit count	1
Canonicalized	Yes
Melting point	217 - 218 °C
Boiling point	518.64 °C at 760 mmHg
Solubility	Soluble in ethanol, DMSO, and ether
Appearance	Pale yellow powder
